# Seroprevalence and risk factors of brucellosis among slaughtered indigenous cattle, abattoir personnel and pregnant women in Ngaoundéré, Cameroon

**DOI:** 10.1186/s12879-018-3522-x

**Published:** 2018-12-03

**Authors:** Julius Awah-Ndukum, Mohamed Moctar Mouliom Mouiche, Lucy Kouonmo-Ngnoyum, Houli Nicolas Bayang, Tanyi Kingsley Manchang, Rodrigue Simonet Namegni Poueme, Justin Kouamo, Victor Ngu-Ngwa, Emmanuel Assana, Kameni Jean Marc Feussom, André Pagnah Zoli

**Affiliations:** 1 School of Veterinary Medicine and Sciences, University of Ngaoundéré, Ngaoundéré, Cameroon; 2 Department of Animal Science, Faculty of Agronomy and Agricultural Sciences, University of Dschang, Dschang, Cameroon; 3 Institute of Agricultural Research for Development, Veterinary Research Laboratory, Wakwa Regional Center, Ngaoundéré, Cameroon; 4 National Veterinary Laboratory, Garoua, Cameroon; 5 Epidemio-Surveillance Service, Ministry of Livestock, Fisheries and Animal Industries Yaoundé, Yaoundé, Cameroon

**Keywords:** Brucellosis, Cattle, Humans, Prevalence, Risk factors, Ngaoundéré-Cameroon

## Abstract

**Background:**

Brucellosis is a neglected debilitating zoonosis with a high prevalence in many developing countries. Bovine brucellosis is widespread in Cameroon but the epidemiological situation of human brucellosis is not known. A cross sectional study was carried to determine the seroprevalence and factors associated with bovine and human Brucellosis among abattoir personnel and pregnant women in Ngaoundéré, Cameroon.

**Methods:**

Serum sample from 590 abattoir cattle and 816 plausible occupational risk and vulnerable humans to brucellosis (107 abattoir personnel and 709 pregnant women) were collected and screened for anti-brucella antibodies using Rose Bengal Plate Test (RBPT) and ELISA tests. Structured questionnaires were used to collect data on socio-demographics and risk-factors. The differences in proportions between seropositive and seronegative reactors were tested using odds-ratio and χ^2^tests.

**Results:**

Bovine brucellosis seroprevalence was at 3.40% (*n* = 590; 3.4% for RBPT, 5.93% for i-ELISA). Human Brucella seroprevalence was at 5.6% among abattoir personnel (*n* = 107; 5.6% for RBPT, 12.15% for Brucella IgG ELISA) and 0.28% in pregnant women (*n* = 709; both tests). Breed (*P* < 0.00001) was associated with increased risk of brucellosis in cattle and the seroprevalence was highest among the Djafoun (OR = 16.67, 95%CI: 4.49–28.85) and Akou (OR = 16.96, 95% CI: 0.10–23.91) cattle compared to the other breeds. There was a moderate positive correlation (R^2^ = 0.5025) of *Brucella* IgG concentrations (> 200 U/ml) and clinical data for Brucella IgG ELISA seropositive humans. Several potential factors were associated (*P* > 0.05) with increased risk of human brucellosis seroprevalence among the abattoir personnel. The abattoir personnel were essentially males; the seropositive respondents were male and did not use protective equipment at work. Handling of foetus and uterine contents (OR = 13.00, 95%CI: 1.51–111.88) was associated with increased risk of human brucellosis.

**Conclusions:**

Antibrucella antibodies are prevalent in cattle (3.40%), among abattoir personnel (5.60%) and in pregnant women (0.28%) in Ngaoundéré, Cameroon. The study reports the first evidence of human brucellosis in Cameroon and therefore, an indication of a real public health problem. Public awareness campaigns and health education especially among livestock professional and in agropastoral communities should be highlighted to disseminate knowledge, associated risk factors and control measures of brucellosis.

## Background

Brucellosis is an infectious disease of many animal species and humans caused by bacteria of the genus *Brucella* [1] and characterized by inflammation of the genitals and foetal membranes, abortions, sterility and lesions in the lymphatic system and joints [2–7]. Brucellosis is an anthropozoonosis which cause great economic losses in livestock production and seriously threatens public health in countries where it is endemic [1, 7–10]. Human brucellosis has been associated with acute febrile illness, severe debilitating disease that requires prolonged treatment with a combination of antibiotics, permanent disabling sequel, considerable medical expenses and loss of income due to loss of working hours [11, 12]. Spontaneous miscarriage and in utero foetal death during the first trimesters have also been reported among pregnant women [13]. The risks of zoonotic transmission of the disease from animals to humans are associated to climate change and corollaries of husbandry practices, eating habits and social behaviour of the populations concerned [14].

Animal and human brucellosis is endemic and neglected in Sub-Saharan Africa [15] due to lack of attention and absence of adequate diagnostic facilities [14, 16], lack of public awareness, inadequate public-sector animal health services, and poor or low-income communities [16, 17]. However, the prevalence of risk factors for infections are better understood for brucellosis in domestic ruminants particularly bovine brucellosis and this species bias is reflected in control activities [16]. Nonetheless, the surveillance of bovine brucellosis is generally poor and mass control is difficult to implement due to the existence of conditions that favour the widespread nature and transmission of the disease in most of the region [18, 19]. These factors include uncontrolled animal movement, migrations of pastoralists in search of pasture and water, purchase of infected cattle from livestock market for replacement or upgrading, anarchic development of urban livestock breeding and nature of the animal production system, inadequate sanitary measures, demographic factors, regulatory issues, climate, deforestation and wildlife interaction [20–24].

Although there is great progress in controlling brucellosis in some countries, the disease still persists in domestic animals in many regions with frequent transmission to human populations and occurrence of human disease [6]. The geographical distribution of zoonotic brucellosis is strongly correlated with regions where livestock is the main source of human livelihood such as food and income [5]. Brucellosis is an important human disease in the Mediterranean countries of Europe, Africa, Middle East, South and Central Asia and Central and South America [6, 12, 25] and yet it is neglected, underrecognized and frequently goes unreported [6, 12, 25]. Human brucellosis is endemic in Sub-Saharan Africa and seroprevalence estimates have been reported for many countries including 3.8% in Chad [1], 3.3% in Central African Republic, 7.7% in Tanzania [11], 24.1% [26] and 31.82% [27] in Nigeria, 17% in Uganda [28] and 1–5.6% among traditional pastoralists (Fulani) and 0–1.6% among non-pastoraalists in Togo [15].

There is an operational and functional “One Health” National Strategy as well as a National Program for the prevention and control of emerging and re-emerging zoonoses in Cameroon. The “One Health” National Strategy evolved from the combined efforts of sectors of animal health, human health and environmental health working jointly in a trans-sectoral and synergic manner for the management of health security of animal and human population [29]. The National Program for the Prevention and Fight against Emerging and Re-Emerging zoonoses was elaborated with the support of the RESPOND project – USAID [30] and, in 2014, a National Program for the prevention and control of emerging and re-emerging zoonoses was enacted in Cameroon. Using inputs from the human health, livestock, environment, wildlife, research, and higher education sectors and tools developed by the U.S. Centers for Disease Control and Prevention (CDC) five priority zoonotic diseases were identified as from a list of relevant zoonoses for Cameroon including rabies, anthrax, highly pathogenic avian Influenza, Ebola and Marburg Virus disease, and bovine tuberculosis [31]. However, poor implementation of essential control measures of zoonoses including animal brucellosis (e.g., restricting movement of infected cattle, reporting disease to the veterinary services, testing of animals) has been reported in Cameroon [32]. Brucellosis is an important notifiable disease worldwide and there is dearth of information on the epidemiological situation of human brucellosis in the country particularly the seroprevalence of brucellosis among vulnerable communities and populations at risk including abattoir personnel and pregnant women in the Adamawa region which is the main livestock producing region of Cameroon. There are little or no concerted veterinary and medical efforts to maximize brucellosis detection rates. Active involvement of populations at risk and good health systems are lacking such that appropriate preventive measures and planning for effective control programs of brucellosis in animals and humans cannot be achieved [33]. Bovine brucellosis is widely endemic in Cameroon and prevalence rates in the range of 3–31% in cattle at individual levels and 16.2–35.0% at herd levels have been reported [2, 10, 22, 33–38]. However, determining the prevalence and risk factors of brucellosis in all livestock according to their origin could improve the epidemiology the disease in Cameroon. There are also concerns about brucellosis in other farm animals such as sheep, goats and pigs since the occurrence and epidemiology of the disease in these animals is poorly understood. Furthermore, the zoonotic potential and status of brucellosis in human communities as well as the relation between the burden and associated risk factors of brucellosis in livestock and livestock professionals in major livestock procuring zones in the country are not known.

Therefore, this study was carried out to contribute to the epidemiology of bovine and human brucellosis and estimate the seroprevalence of brucellosis in slaughtered cattle, abattoir personnel and pregnant women in Ngaoundéré Cameroon. The study also assesses the risk factors for evidence-based control of the disease in Cameroon**.**

## Methods

### Description of study areas

**Fig. 1:**
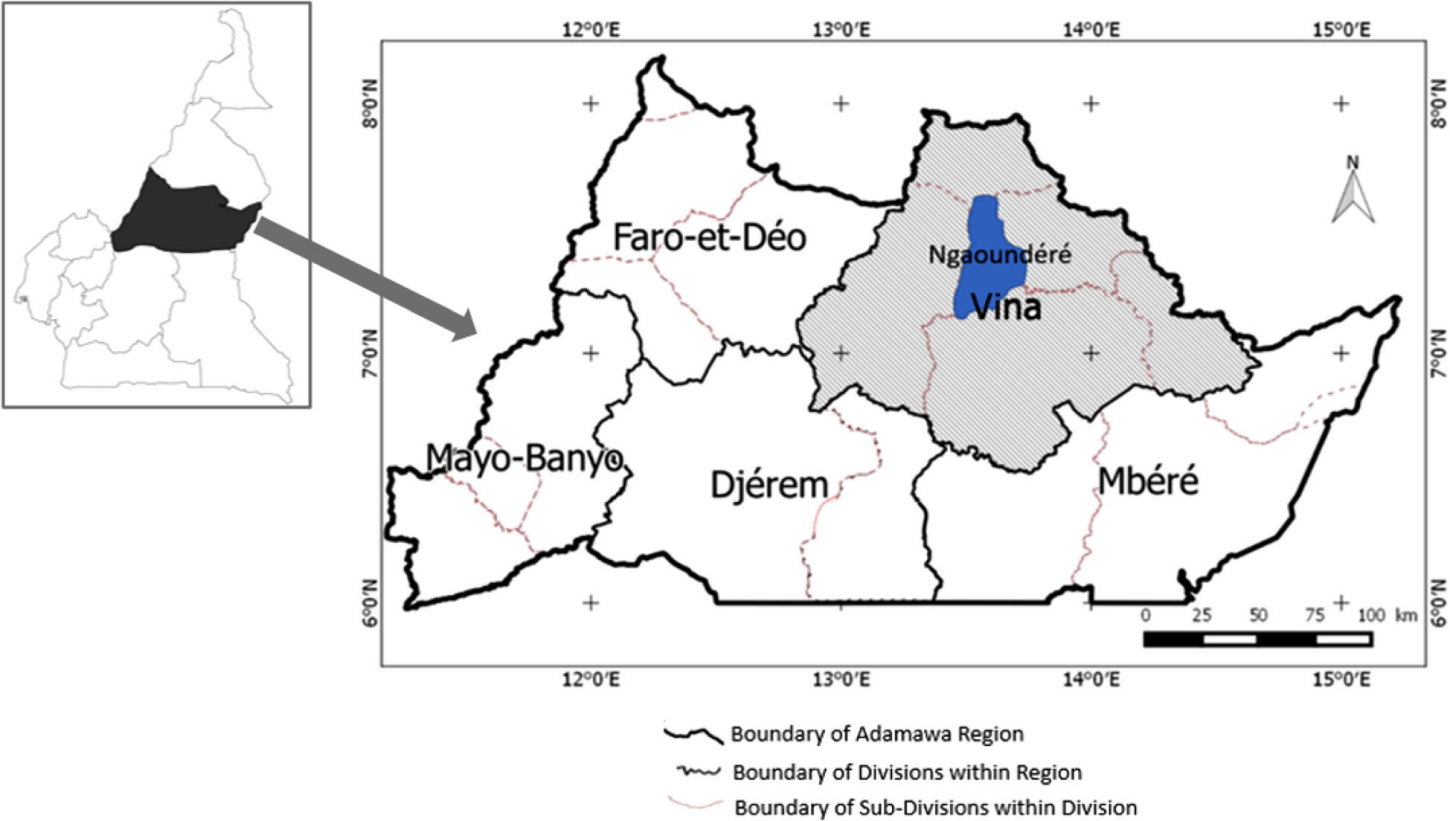
Map showing study area (Ngaoundéré) in Vina Division of the Adamawa Region in Cameroon. (Source: Ngaoundéré City Council for map of Adamawa Region, Cameroon. Map of Cameroon was adapted from Wikimedia Commons: https://commons.wikimedia.org/wiki/Maps_of_Cameroon)

The study was carried out during the period of August 2015 to March 2016 in Ngaoundéré (7°09′ – 7°70’N and 13°52′ - 13°70′E) in Vina Division of the Adamawa Region in Cameroon (Fig. 1). The Adamawa region is located in the Savannah Guinean highland, in the mid to high altitude zones of Cameroon with an annual precipitation of 1200–1600 mm, rainy season from mid-March to October and temperature 23–25 °C [39]. The region is a major cattle production zone in the country and major beef cattle supplier to the southern zones [40]. The communities of the study areas are mainly pastoralists (30%) and agropastoralists (65%) and practice predominantly the traditional systems of husbandry. The socioeconomic, political, cultural, and religious activities of the communities are dependent on crop production and keeping of livestock including cattle, sheep, goats, pigs and poultry. *Bos indicus, Bos Taurus* (Namchi), and exotic (Montbeliarde, Holstein, Charolaise) breeds of cattle as well as their crossbreeds are reared in the study areas.

### Selection of animals for the study

Selection of individual cattle for the study was done in the Ngaoundéré municipal abattoir during the study period using previously described systematic random sampling technique [41]. An individual prevalence rate of 5.40% [33] was used to estimate the sample size as described by Thrusfield [42]. Briefly, about 20% of 40–60 cattle slaughtered daily in the abattoir was randomly selected each day, except on Saturday and Sunday, were included in the study. Based on a calculated sampling fraction of five (every fifth animal was sampled) for daily use, the first animal was selected by picking one animal by random generation method of the first five animals on the slaughter chain. Thereafter, every fifth animal (adding 5 to previous picked number) was chosen till the sample size was achieved. Information related to the breed, sex, age and body condition score of the animal were noted. Estimation of ages was done by dental inspection and examination of horn rings for animals without teeth (especially old/adult females) while the breeds of the animals were obtained as previously described [43–46]. The body condition score was done by assessing the general appearance and palpation of the lumbar region of the animal on a scale of 1 to 5 and further classed into 3 categories: 1–2 (poor), 3 (good) and 4–5 (very good) as previously described [47].

### Selection of human samples for the study

The targeted human populations were cattle professionals and plausible vulnerable humans who have contacts with livestock as well as consumers of fresh beef and dairy products in Ngaoundéré city and the environ. However, abattoir personnel (persons who had administrative- and meat-activities in the Ngaoundéré municipal abattoir) and pregnant women on antenatal consultations and women with recent history of miscarriages at the Obstetrico-gynaecological unit of the Ngaoundéré Regional Hospital were plausible candidates for the study. All personnel within the abattoir premise were contacted while the sample size of the pregnant women was estimated as previously described [48]. Briefly, the humans sampled were 107 of 120 persons that were present in the vicinity of the Ngaoundéré abattoir (including abattoir workers (butchers, butcher apprentices, meat sellers, veterinary inspectors, administrative staff), cattle owners and cattle traders that visited the abattoir, restaurant vendors at the abattoir to purchase meat) and 709 pregnant women (predominantly from Ngaoundéré and its environs) on antenatal consultations at the Ngaoundéré Hospital during the study period.

### Blood sampling and laboratory analysis

Apart from procedural restraining manipulations for safety purposes and jugular venipuncture for blood sampling (≥5 ml) using sterile vacutainer, the animals were not subjected to suffering. Blood samples were collected from the chosen animals on the chain before slaughter. Serum samples were extracted from collected blood and stored at − 20 °C until laboratory analysis at the Veterinary Research Laboratory of the Institute of Agricultural Research for Development, Wakwa Regional Center, Ngaoundéré, Cameroon. The state of gravidity was determined by the presence or absence of foetus in uterus following evisceration of slaughtered cows.

Human blood samples were collected by a team of two nurse practitioners recruited for the purpose. Apart from procedural medical manipulations, blood (≥5 ml) was collected at the level of the median and cephalic veins using sterile vacutainer, the humans were not subjected to discomfort. Following sampling, specific details of the participant was noted to avoid blood collection from same persons on later antenatal consultations. Serum samples were extracted from collected blood and stored at − 20 °C until laboratory analysis at the Bacteriological Laboratory of the Ngaoundéré Regional Hospital, Cameroon.

### Serological tests

Following Rose Bengal Plate test (RBPT) screening of all cattle (590 serum) and human [812 serum (107 persons on abattoir premise and 705 pregnant women)] samples, Enzyme-Linked Immunosorbent Assay (ELISA) was performed on the cattle and human samples to detect anti-brucella antibodies. Each batch of test was included with a positive control and a negative control. A subject was seropositive when the serum tested positive to RBPT and or ELISA.

#### Rose Bengal Plate Test

RBPT was performed as described by Alton et al. [49]. Briefly, the sera and antigen were brought to room temperature before use. Equal volumes (30 *μ*L) of standardized *B. abortus* antigen Weybridge strain 99 and test serum were mixed thoroughly and rotated on a glass plate using a stick applicator, and the plate was rocked for 4 min. The appearance of agglutination, recorded as positive, within 1 min was scored 4+ (++++) and between 1 and 4 min was scored 1+ to 3+ (+, + +, and + + +) according to the different degrees of agglutination. The absence of agglutination within 4 min was regarded as negative (−).

#### Detection of brucellosis antibodies in cattle

Commercial indirect multispecies ELISA (i-ELISA) (ID.Vet, Innovative Diagnostics, France) for the detection of antibrucella (*B. arbortus, B. melitensis and B. Suis*) antibodies in the cattle serum was performed according to the manufacturer’s instructions and essentially as described by Limet et al. [50]. The test was conducted in 96-well polystyrene plate that was precoated with purified Brucella *abortus* lipopolysaccharide (LPS) antigen. An anti-multi-species-IgG horseradish peroxidase (HRP) was used as conjugate as described by Saegerman et al. [51]. The substrate solution (TMB + DMSO + H_2_O_2_) was added after washing to eliminate excess conjugate. The coloration of antigen-antibody conjugate-peroxidase complex formed depended on the quantity of anti-*Brucella* antibodies that was present in the specimen tested. Thus, in the presence of antibodies, a blue solution appeared which became yellow after addition of the stop solution, while in the absence of antibodies, no coloration appeared. The optical density (OD) of the well was read at 450 nm by an automatic micro plate reader and for each sample *S*/*P*% was calculated [1] as follows:

$$ \frac{S}{P}\%=\frac{\left({OD}_{sample}-{OD}_{nc}\right)}{\left({OD}_{pc}-{OD}_{nc}\right)}x100 $$

where OD_sample_, OD_nc_, and OD_pc_ are the readings of optical densities for the sample, negative control, and positive control, respectively. The samples were classified as positive if *S*/*P*% ≥120%, negative if *S*/*P*% ≤ 110%, and doubtful if 110% < *S*/*P*% < 120%. Also, the fact that OD_pc_ > 0.350 and OD_pc_/OD_nc_ > 3 indicated that the tests were working properly.

#### Detection of brucellosis antibodies in humans

The commercial Brucella IgG ELISA kit (RE56841®, IBL International GMbH, Hamburg, Germany) for qualitative and quantitative determination of IgG antibodies against *Brucella* in human serum was performed according to the manufacturer’s instructions and essentially as described by Esmaeili et al. [52]. Briefly, the ELISA was based on the sandwich principles. The wells were coated with antigen and specific antibodies of the sample binding to the antigen coated wells were detected by secondary enzyme conjugated antibody specific for human IgG (horseradish peroxidase-conjugated anti-human IgG). After tetra methyl benzidine (TMB) substrate reaction, a Brucella antibody-antigen reaction was indicated by a blue coloration. The intensity of the blue coloration that developed proportional to the quantity of IgG-specific antibodies detected. The optical density (OD) of the well was read at 450 nm by an automatic micro plate reader. Positive, negative and cut-off controls were included in the test. Antibody activities were calculated using a standard curve according to the manufacturer’s guidelines. However, the Cut-off value was obtained from the optical density (OD) of the Cut-off control and the Cut-off index (COI) was calculated from the optical densities of the sample and Cut-off value as follows:

$$ COI\%=\frac{OD_{Sample}}{OD_{Cut- off\  control}}x100 $$

where OD_Sample_ and OD_Cut-off control_ are the readings of optical densities for the sample and cut-off control, respectively. The samples were classified as positive if COI % ≥120%, negative if COI % ≤ 80%, and doubtful if 80% < COI % < 120%.

The samples were classified quantitatively as positive if IgG concentration [IgG] ≥1.2 U/mL, negative if [IgG] ≤ 0.8 U/mL, and doubtful if 0.8 U/mL < [IgG] < 1.2 U/mL. The quantitative results were correlated to clinical data recorded during questionnaire interview survey.

### Risk factor analysis

Information on risk factors for bovine and human brucellosis was obtained by examination of individual cattle as well as questionnaire interview of personnel at the Ngaoundéré abattoir and pregnant women on antenatal consultations at the Ngaoundéré Hospital. The questionnaires were structured to collect information on a range of variables including lifestyle, socio-demographic data, clinical history related to brucellosis and awareness of zoonotic brucellosis.

### Ethical consideration

Risk assessments of the project were performed by the researchers to avoid hazards to all persons and animals involved in the project. Permission for the study was obtained from the required authorities and Local Ethical Committees in Adamawa Region, Cameroon including the Regional delegation of Livestock, Fisheries and Animal Industries, Regional Delegation of Public Health, School of Veterinary Medicine and Sciences of the University of Ngaoundéré and Ngaoundéré Regional Hospital. The purpose of the study was explained (with the assistance of local veterinary and medical practitioners, community leaders and trusted intermediaries) to personnel at the Ngaoundéré abattoir and pregnant women at the Ngaoundéré hospital. Cattle professional including butchers (for animal survey), personnel at the Ngaoundéré abattoir and pregnant women on antenatal consultations at the Ngaoundéré Hospital (for human survey) were used in the study after giving their written informed consent.

### Data analysis

The data were analysed using “R” software (@Manual {, title = {R: A Language and Environment for Statistical Computing}, author = {{R Core Team}}, organization = {R Foundation for Statistical Computing}, address = {Vienna, Austria}, year = {2018}, url = {https://www.R-project.org/},}) and reduced to percentiles. The chi-square test was used to test significant levels within factors on seroprevalence rates and odds-ratios were determined for associated risk factors along 95% confidence intervals and statistical significance set at *P*< 0.05.

## Results

### Seroprevalence rates of bovine and human brucellosis in Ngaoundéré

Combination of tests results of 590 abattoir cattle revealed an overall apparent seroprevalence of 20 (3.40% [1.94–4.86]) with 20 (3.40% [1.94–4.86]) for RBPT and 35 (5.93% [4.03–7.83]) for i-ELISA (Table 1). For the human study, the tests results of 107 abattoir personnel gave an overall apparent seroprevalence of 6 (5.60% [1.24–9.96]) with 6 (5.60% [1.24–9.96] for RBPT and 13 (12.15% [5.96–18.34]) for Brucella IgG ELISA (Table 1). The tests results showed that 2 (0.28% [1.25–9.95]) of 709 sampled pregnant women in the Ngaoundéré Hospital were both RBPT and Brucella IgG ELISA seropositive to brucellosis.

**Table 1:** Brucellosis seropositivity among slaughtered cattle (*n* = 590) and personnel (*n* = 107) in Ngaoundéré municipal abattoir of Cameroon according to combined results of Rose Bengal Plate test and Enzyme Linked Immunosorbent Assay

Serological results	Number of cases (% [95% CI])
Cattle (*n* = 590)	
RBPT (+)	20 (3.40 [1.94–4.86])
RBPT (−)	570 (96.60 [95.14–98.06])
i-ELISA (+)	35 (5.93 [4.02–7.84])
i-ELISA (−)	555 (94.07 [92.16–95.98])
RBPT (+) i-ELISA (+)	20 (3.40 [1.94–4.86])
RBPT (+) i-ELISA (−)	0
RBPT (−) i-ELISA (+)	15 (2.54 [1.27–3.81])
Abattoir personnel (*n* = 107)	
RBPT (+)	6 (5.60 [1.24–9.96])
RBPT (−)	101 (94.40 [90.04–98.76]
Brucella IgG ELISA (+)	13 (12.15 [5.96–18.34])
Brucella IgG ELISA (−)	95 (87.85 [81.66–94.04]
RBPT (+) Brucella IgG ELISA (+)	6 (5.60 [1.24–9.96]
RBPT (+) Brucella IgG ELISA (−)	0
RBPT (−) Brucella IgG ELISA (+)	7 (6.54 [1.86–11.22])

(−): negative; (+): positive; *RBPT* Rose Bengal Plate test, *ELISA* Enzyme linked immunosorbent assay, *i-ELISA* Indirect enzyme linked immunosorbent assay

The overall occurrence of brucellosis seropositivity among abattoir personnel and cattle revealed that the presence of brucellosis in cattle presents a non-negligible risk for the disease in humans.

The study revealed a moderate positive correlation (R^2^ = 0.5025) of clinical symptoms and *Brucella* IgG concentrations in Brucella IgG ELISA seropositive humans. Overall, 10 (66.67%) of 15 seropositive humans (13 butchers and 2 pregnant women) reported non-specific clinical symptoms during the study period including fever, asthenia (abnormal body weakness), arthralgia (painful joints), myalgia (muscular pain) and excessive sweating during the study and presented > 200 U/ml (range: 250–350 U/ml) serum *Brucella* IgG concentrations. However, 5 (33.33%) seropositive humans, who were males abattoir personnel, reported milder symptoms in various combinations (fever (03), asthenia (body weakness) (03), arthralgia (painful joints) (02), myalgia (Muscular pain) (02) and or sweating (02)) and showed < 50 U/ml (range: 15–30 U/ml) serum *Brucella* IgG concentrations.

### Factors affecting seroprevalence of bovine brucellosis in Ngaoundéré municipal abattoir

The study revealed that significantly higher seroprevalence rates were recorded for the Djafoun (16.67% [4.49–28.85]) and Akou (16.96% [0.10–23.91]) cattle compared to the other breeds in the area (Table 2). Calculation of odds-ratio showed that the Djafoun (9.40 [3.06–28.87]) and Akou (9.60 [4.08–22.62]) were over 9.4 times more likely (*P* < 0.05) than it was for the Bokolo (3.92 [0.45–33.86]) and cross (1.07 [0.13–8.74]) breeds (*P* > 0.05) compared to the Gaudali breed to be anti-brucella seropositive.

**Table 2:** Brucellosis seropositivity among slaughtered cattle (*n* = 590) in Ngaoundéré municipal abattoir of Cameroon according to risk factors

Category	Variable	Number^a^ (Positive)	Seropositivity using i-ELISA % [95%CI]	*P*-value (χ^2^)
Breed	Gudali	384 (8)	2.08 [0.01–3.51]	< 0.00001* (43.2371)
	Bokolo	13 (1)	7.69 [0–22.17]	
	Djafoun	36 (6)	16.67 [4.49–28.85]	
	Akou	112 (19)	16.96 [0.10–23.91]	
	Cross-breed^b^	45 (1)	2.22 [0–6.52]	
Sex	Female	529 (32)	6.05 [4.02–8.08]	0.7232 (0.1254)
	Male	61 (3)	4.92 [0–10.34]	
Age (years)	Young (< 4)	60 (2)	3.33 [0–7.87]	0,3095 (2.3453)
	Adult (4–8)	356 (19)	5.34 [3.00–7.68]	
	Old (> 8)	174 (14)	8.05 [4.00–12.09]	
Body Condition Score	Poor (< 3)	130 (13)	10.0 [4.84–15.16]	0.0828 (4.9807)
	Good (3–4)	412 (20)	4.85 [2.78–6.92]	
	Very Good (> 4)	48 (2)	4.17 [0–9.82]	
State of gravidity	Pregnant	185 (11)	5.95 [2.54–9.36]	0.9418 (0.0053)
	Non-pregnant	344 (21)	6.10 [3.57–8.63]	

^a^Observed reactions of individual animals (*n* = 590) or of animals whose data where noted (*n* depends on number of animals e.g. gravidity) in the category

^b^Crossbreed between local breeds

*Significantly different (*P* < 0.05)

Sex, age, body condition score and state of pregnancy had no significant effect (P > 0.05) on the seroprevalence of bovine brucellosis in this study.

### Factors affecting brucellosis seroprevalence in personnel of the Ngaoundéré abattoir

The rate of *Brucella* IgG seropositive reactions among the abattoir personnel varied according to the lifestyle and activities of the different categories of respondents (Table 3). *Brucella* IgG seropositive respondents were essentially male. Non-significantly higher (*P* > 0.05) rates associated with age, poor educational level, contact with non-abattoir animals and post of activity at the abattoir were observed. However, 20 abattoir personnel (including veterinary inspectors, administrative staff, cattle owners and cattle traders that visited the abattoir and restaurant vendors at the abattoir to purchase meat) as well as 04 personnel with post-secondary education were seronegative to brucellosis.

**Table 3:** Socio-demographic characteristics and brucellosis seroprevalence among personnel (*n* = 107) of the Ngaoundéré municipal abattoir in Cameroon

Characteristics		Number (positive)	Seropositivity using Brucella IgG ELISA % [95%CI]	P-value (χ^2^)
Sex	Female	11 (0)	0.00	–
	Male	96 (13)	13.54 [6.70–20.39]	
Age (years)	[15–25]	20 (1)	5.00 [0–14.55]	0.732 (1.289)
	[25–35]	47 (6)	12.77 [3.23–22.31]	
	[35–45]	26 (4)	15.38 [1.52–29.25]	
	[45–65]	14 (2)	14.29 [0–32.62]	
Education level	None	13 (3)	23.08 [0.17–45.98]	0.273 (2.599)
	Primary	48 (7)	14.58 [4.60–24.57]	
	Secondary	42 (3)	7.14 [0–14.93]	
Marital status	Married	69 (10)	14.49 [6.19–22.80]	0.317 (0.9995)
	Unmarried	38 (3)	7.89 [0–16.47]	
Duration of working at the abattoir (Years)	Years ≤5	33 (2)	6.06 [0–14.20]	0.368 (3.1595)
	5 < Years≤10	22 (2)	9.09 [0–21.10]	
	10 < Years≤20	44 (7)	15.91 [5.10–26.72]	
	Years > 20	08 (2)	25.00 [0–55.01]	
Post occupied / activity in the abattoir^a^	Handle foetus and uterine contents	6 (3)	50.00 [9.99–90.01]	0.067 (7.173)
	Clean offal	12 (2)	16.67 [0–37.75]	
	Meat seller	41 (6)	14.63 [3.8–25.45]	
	Slaughter and dress animals (Butcher)	28 (2)	7.14 [0–16.68]	
Have cattle at home	Yes	46 (5)	10.87 [1.87–19.86]	0.725 (0.124)
	No	61 (8)	13.11 [4.64–21.59]	
Have contact with carnivores	Yes	8 (1)	12.50 [0–35.42]	0.975 (0.001)
	No	99 (12)	12.12 [5.69–18.55]	
Have contact with sheep and goats	Yes	47 (6)	12.77 [3.23–22.31]	0.863 (0.030)
	No	60 (7)	11.67 [3.54–19.79]	
Have contact with pigs	Yes	4 (0)	0.00	–
	No	103 (13)	12.62 [6.21–19.03]	

^a^ Butchers and Butcher apprentices

The potential risk factors that may be attributed to the occurrence of brucellosis among personnel of the Ngaoundéré abattoir are presented in Table 4. All *Brucella* IgG seropositive respondents did not use of personal protective equipment (such as gloves) during work. Non-significantly higher (*P* > 0.05) rates associated with longevity in the abattoir environment, activity at the abattoir, exposure to animals outside abattoir and home environments, consumption of raw milk and lack of knowledge about brucellosis were the potential factors for the *Brucella* IgG seropositive reactions observed. However, personnel who handled foetus and uterine contents were significantly affected compared to butchers (*P* < 0.01, χ^2^ = 7.24) and meat sellers (*P* < 0.04, χ^2^ = 4.23) at the abattoir. Calculation of odds ratio showed that personnel who handled foetus and uterine contents were 13.00 (1.51–111.88) times more likely (*P* < 0.05) than it was for the cleaners of offal (2.60 [0.32–21.05]) and meat sellers (2.23 [0.42–11.94]) (P > 0.05) of being *Brucella* IgG seropositive compared to butchers.

**Table 4:** Brucellosis seropositivity among personnel of the Ngaoundéré municipal abattoir according to potential risk factors (*n* = 107)

Variable		Number (positive)	Seropositivity using Brucella IgG ELISA % (95%CI)	Odds ratio (95%CI)	*P*-value
Animal exposure at home	Yes	46 (5)	10.87 (1.87–19.86)	1	0.4833
	No	61 (8)	13.11 (4.64–21.59)	1.24 (0.38–4.07)	
Consume raw milk	Yes	49 (9)	18.37 (7.53–29.21)	3.04 (0.87–10.57)	0.0650
	No	58 (4)	6.90 (0.38–13.42)	1	
Use of protective equipment at work	Yes	13 (0)	0	–	–
	No	92 (13)	14.13 (7.01–21.25)		
Manipulate with aborted fœtus	Yes	23 (3)	13.04 (0–26.81)	1.11 (0.28–4.42)	0.5625
	No	84 (10)	11.90 (4.98–18.83)	1	
Knowledge of brucellosis	Yes	12 (1)	8.33 (0–23.97)	0.63 (0.07–5.32)	0.5547
	No	95 (12)	12.63 (5.95–19.31)	1	
Animal exposure outside the abattoir and home	Yes	59 (8)	13.56 (4.82–22.30)	1.38 (0.42–4.53)	0.4096
	No	49 (5)	10.20 (1.73–18.68)	1	
Longevity at the abattoir (years)	≤ 5	33 (2)	6.06 (0–14.20)	0.37 (0.08–1.77)	0.1675
	≥ 5	74 (11)	14.86 (6.76–22.97)	1	

However, two seropositive pregnant housewives with no formal occupation who had regular contact with domestic ruminants (cattle, sheep and goats), regularly consumed unpasteurised milk, assisted in dressing of slaughtered animals and manipulated aborted foetuses and other uterine contents without using personal protective equipment such as gloves were observed in the study. Both women were in the range of 35–45 years old, had suffered miscarriages in the past and were in the second and third trimester of pregnancy respectively.

## Discussion

The overall seroprevalence of bovine brucellosis obtained at the Ngaoundéré municipal abattoir (3.4% for RBPT and 5.93% for i-ELISA) is different from the rates reported in other parts of the country. Though several other studies reported higher bovine brucellosis seroprevalence ranging from 7 to 31% in various parts of Cameroon [22, 33–38, 53], lower seroprevalence rates have been recorded in indigenous cattle such as 3% using competitive ELISA [37] in Adamawa Region and 4.6% with RBPT in Northwest region [36]. The results obtained in this study is similar to various serological findings reported in indigenous cattle farming systems in Niger (1.3%) [54], Ivory Coast (4.6%) [55], Nigeria (3.9%) [24], Chad (2.6%) [1, 56], Central Africa Republic (3.3%) [57], Uganda (3.3%) [58], Zimbabwe (5.6%) [59, 60], and Ethiopia (2.4–3.9) [23, 61, 62] as well as in the municipal cattle slaughterhouse (4.88 and 5.82%) in Ibadan Nigeria [27]. However, higher rates have been reported in Ivory Coast (8.8–10.3%) [63, 64], Zambia (18.7%) [20], Mali (22%) [65], Burkina Faso (13.2%) [66], and Algeria (9.7%) [67]. The differences in prevalence rates reported in Cameroon and other parts of Africa could also be associated with the evolution of the disease, geographical origin, breeds, sample size, study frame as well as the protocol adopted such as the type and number of diagnostic tests used. The protocol could have involved one test or more than one test in series (screening test followed by confirmation of positive reactors by another test) or in parallel (all tests are applied on the sampled animals independently) [33, 55, 63, 68–70]. Furthermore, close antigenic cross-reactivity with other bacterial infections *(Yersinia*, *Xanthomonas*, *Salmonella*, *Streptococci*, *E. coli, tuberculosis)* can lead to false positive results being encountered in serological diagnosis of brucellosis [71, 72].

The study observed that breed was the major factor for high bovine brucella seropositivity compared to sex, age, body condition score and state of gravidity of the animals that had no significant influence on the seroprevalence. The finding is similar to Akinseye et al. [24] and Ojong [36] who did not observe differences in seropositivity due to sex. It is contrary to Ojong [36] who reported difference due to breed and Awah-Ndukum et al., [33] who reported differences due to age and sex and not by breeds and body condition score. Though level of susceptibility of breed to brucellosis was not ascertained by the study, the difference observed are associated to the ethnic groups of pastoral communities have different behaviours in conducting and systems of keeping their livestock. The Djafoun and Akou cattle in this study are kept by the Mbororo / Fulani ethnic groups who predominantly associate transboundary animal movements, migrations and transhumance to their husbandry activities compared to the Foubles who keep Gudali cattle and are generally sedentary. Domenech et al., [2] found a brucellosis seroprevalence ranging from 15 to 40% in cows in pastoral/agropastoral systems in Chad and Cameroon, which mixed up all animals (pregnant or not) compared to brucellosis seroprevalence of 8.5% in cattle of a particular tribe / ethnic group who kept their cattle in small herds during the rainy season and grouped all the animals together during the dry season to move to graze land with the exception of pregnant animals which remained in the village. Also, the major source of variation for brucellosis prevalence in the of risk of different diseases was observed between-farm [73], suggesting that cattle herd management practice within production systems could be more important factors than the mainly environmental variables used for differentiating between the systems [2, 74]. General poor condition of animals [75], aging and high parity [3, 4, 14, 34, 66, 76] have been observed to significantly increase bovine brucellosis seroprevelance. In addition, animals become more sensitive to brucella infection at reproductive age [34, 76, 77].

Though brucellosis occurs naturally in animals, the human disease has been reported especially in regions where bovine brucellosis is endemic [6] and its prevalence in humans tends to correspond to that in animals [6, 28, 78]. In the present study, the overall brucellosis seroprevalence among abattoir personnel (5.6%) is comparable to the overall seroprevalence of bovine brucellosis at the abattoir (3.4%) and 3% rate earlier reported in live animals using complement ELISA [37]. However, Brucella IgG ELISA brucella seropositivity (12.15%) among abattoir personnel was significantly higher than i-ELISA brucella seropositivity (5.93%) in the abattoir cattle. The high Brucella IgG ELISA human seroprevalence parallels with bovine brucellosis seroprevalence (RBPT, i-ELISA, competitive ELISA) reported in the study region and other parts of Cameroon which ranged from 7 to 31% in live cattle [33–35, 38]. The seropositive humans (> 66.67%) presented > 200 U/ml IgG concentrations and also reported febrile illnesses, body weakness, sweating, painful joints and muscular pains. Seropositive pregnant women in the study regularly consume raw milk and had history of exposure to aborted animal foetuses and previous miscarriages. Brucellosis patients have been associated with significantly elevated levels of *Brucella* IgG and differentiation between brucellosis from non-brucellosis patients have been done by measuring *Brucella* IgG concentrations [79]. The finding of *Brucella* IgG seropositive humans with milder clinical symptoms and < 50 U/ml serum *Brucella* IgG concentrations might be due to a long-ago infection with the *Brucella* IgG level waning / decreasing over time and lack of further exposure to infection or source of infection. Sippel et al. [79] stated that ELISA was excellent for screening populations for anti-*Brucella* antibodies and differentiating between phases of the disease and reported high levels of blood IgG which lasted up to 8 months following persisting *Brucella* infections. It is worth noting that these non-specific symptoms (fatigue, myalgias, arthralgias, headaches, chills) are shared by much more prevalent tropical diseases such as malaria [25, 80].

This study presents the first of human brucellosis seroprevalence report from Cameroon and the infected animals in the study area probably serve as reservoirs and sources for the human brucellosis recorded. Therefore, *Brucella* infection is an important public health problem in Cameroon since traditional pastoral and agropastoral communities are widespread with the inhabitants depending almost entirely on livestock for livelihood. The brucellosis seroprevalence (5.6% [5.6% for RBPT, 12.15% for Brucella IgG ELISA]) among abattoir personnel recorded in this study is lower than rates ranging from 10 to 17% among abattoir workers, livestock rearing communities and individuals with febrile illnesses in hospital in Uganda [28, 81, 82], 21.2% among patients with febrile clinical signs, 24.1% among abattoir workers and 44% among butcher workers in Nigeria [26, 83, 84], 40% among pastoralists in Libya [85] and 8% in pastoral communities following implementation of relevant control measures in Egypt [86]. Human brucellosis seroprevalence was usually high among livestock professionals, people who live in pastoral communities, habitually consume raw milk and milk products, in addition to processing milk products [6, 26, 28, 81–88].

This study showed that the human brucellosis seroprevalence varied among the categories of abattoir personnel, suggesting further investigation. Age, poor educational level and longevity of service were associated with slight increase in seroprevalence. Female personnel were brucella seronegative and seropositivity was highest among butcher apprentices who handled foetuses and clean offal, followed by meat sellers and butchers whose main job was slaughtering of animals. Though all seropositive abattoir personnel did not wear protective equipment at work and potential factors were associated with non-significantly higher human brucellosis seroprevalence including consuming raw milk, handling foetuses, occupational exposure of over 5 years, knowledge of brucellosis, owning and contact with livestock outside the abattoir and home environments. However, it should be noted that abattoir personnel in the study were muslim-dominated and may have accounted for the absence of contact between seropositive reactors and pigs. The study revealed that butcher apprentices who handled foetuses and uterine contents were more at risk compared to the other occupational groups probably due of their close contacts with infected blood and tissues of infected animals as well as infected foetuses and uterine contents. Several reports in Nigeria, Tanzania and Egypt have highlighted that among occupational groups in abattoirs, seroprevalence of brucellosis was highest among butchers whose main job was slaughtering of animals, followed by livestock traders, meat sellers and abattoir cleaners compared with the other workers [26, 27, 89, 90]. Also, transmission of human brucellosis by inoculation through cuts and abrasions in the skin [6, 91] and increase of brucellosis seroprevealence among butchers with injuries slaughtering animals compared to other abattoir workers [26] have been reported.

In agreement with the finding of Aworh et al., [26], veterinarians and para-veterinarians, considered to be at high occupational risk, were not found to be *Brucella* seropositive in this study. This may be attributed to their awareness of the zoonotic brucellosis and the use of personal protective equipment and short exposure time of veterinarians at the abattoir during meat inspection coupled with good personal hygiene practices during work. Short exposure time and good personal hygiene practices may also be associated to the *Brucella* seronegative reactions observed among the administrative staff and abattoir visitors (cattle owners and cattle traders that visited the abattoir, restaurant vendors at the abattoir to purchase meat).

There was no statistically significant correlation between human brucellosis seroprevalence and contact with home-owned animals, assisted animal parturitions, slaughter of animals, and contact with domestic animals. However, these findings are contrary to previous reports that the occurrence of human brucellosis was associated with contact with domestic animals [92], exposure to aborted animals and assisting animal parturition [93–95] and or sharing of water sources with animals [96]. Similar to the findings of Tumwine et al., [28], this study largely depended on self-reporting by the participants who could have left out some potential factors associated to zoonotic brucellosis as more seropositive respondents had no knowledge of the disease.

The study used serological tests (RBPT and ELISA) in combination to minimize measurement of false positive errors and revealed that human brucellosis is a real public health problem in Cameroon. No significant association was observed for human brucellosis seropositivity regarding drinking of milk (raw and pasteurized) and knowledge of zoonotic brucellosis (Yes and No). However, emphasizes should be on the importance of drinking only pasteurized milk and sensitization of animal professionals to improve their level of awareness, as the outcome might have been due of the subject group used in the study. Therefore, it is more likely that human brucellosis seroprevalence would be higher in communities where people live among livestock since bovine brucellosis is reported to be highly endemic in the country [22, 33–38].

## Conclusion

Brucellosis is a neglected debilitating zoonosis and an occupational hazard with a high prevalence in many developing countries. Transmission to humans can occur through contact with infected animals and animal products. The study reports the first evidence of human brucellosis in Cameroon and revealed that brucella infection is an important public health problem among abattoir personnel and pregnant women living in Ngaoundéré Cameroon. However, a bacteriological study of brucellosis would be necessary to determine circulating serotypes in the Ngaoundéré area and beyond. Though indigenous cattle were brucellosis seropositive irrespective of sex, age, body condition score and gravidity state, breed was the major factor observed to be associated with bovine brucellosis. The risk of transmission to humans was aggravated by not using protective equipment at work and handling of foetus and uterine contents; and among male abattoir personnel. Public awareness campaigns and health education especially among livestock professional and in agropastoral communities should be highlighted to disseminate knowledge, associated risk factors and control measures of brucellosis. The enlightenment should include discouraging consumption of unpasteurized milk and milk products, encourage animal professionals to consistently use personal protective equipment and good personal hygiene practices at work, regular brucellosis screening and adhering to safe animal-product handling practices. Serological surveillance of human brucellosis and the associated risk factors is essential in Cameroon particularly among livestock professionals and in agropastoral communities. The need for intensification of the integrated “One Health” approach and involving sectoral policies including interdisciplinary strategies between animal and human health experts, concerned target stakeholders and affected communities about the need for detailed information on animal and human brucellosis for effective management in the country cannot be overemphasized.

## Abbreviations

CDC: U.S. Centers for Disease Control and Prevention
COI: Cut-off index
ELISA: Enzyme-Linked Immunosorbent Assay
HRP: Horseradish peroxidase
i-ELISA: Indirect Enzyme Linked Immunosorbent Assay
LPS: Lipopolysaccharide antigen
OD: Optical density
RBPT: Rose Bengal Plate test
TMB + DMSO + H_2_O_2_: Tetramethylbenzidine+Dimethyl sulfoxide+Hydrogen peroxide
